# Associations and Pathways Between Online Health Information–Seeking Behavior and Patient Adherence: Cross-Sectional Study

**DOI:** 10.2196/91115

**Published:** 2026-06-04

**Authors:** Zhenyu Xu, Jingjing Jia, Chaofan Wu

**Affiliations:** 1 Department of Urology, The First Affiliated Hospital of Lishui University, Lishui People's Hospital, Lishui, Zhejiang, China1; 2 School of Medicine and Health Management, Tongji Medical College, Huazhong University of Science and Technology, Wuhan, Hubei, China2; 3 Department of Personnel, The First Affiliated Hospital of Lishui University, Lishui People's Hospital, 15 Dazhong Street, Lishui City, Lishui, Zhejiang, 323000, China, 86 173577109853

**Keywords:** online health information–seeking behavior, patient adherence, physician-patient communication efficacy, digital health platforms, rural residents, health literacy

## Abstract

**Background:**

The widespread adoption of the internet has established online health information–seeking behavior (OHISB) as a primary channel for public health knowledge acquisition, potentially influencing patient adherence behaviors and physician-patient dynamics. However, the underlying pathways, particularly the role of physician-patient communication efficacy and the differential impact of various digital platforms, remain underexplored, especially among rural populations.

**Objective:**

This study examined the association between OHISB and patient adherence among rural residents in China, with a specific focus on the mediating role of physician-patient communication efficacy and the moderating roles of different platform types.

**Methods:**

A cross-sectional survey was conducted from June 2023 to October 2024 using multistage stratified sampling across 6 Chinese provinces. Participants were rural residents aged 18 to 70 years with recent health care experiences. Data from 7004 valid questionnaires were analyzed. A fixed-effects model assessed the primary association, with robustness checked via least absolute shrinkage and selection operator regression. Mediation analysis using the bootstrap method examined the indirect association through physician-patient communication efficacy, and interaction terms tested the moderating effects of platform type (internet hospitals, professional platforms, WeChat accounts, short video apps, and search engines).

**Results:**

OHISB showed a significant positive direct association with patient adherence (β=0.260; *P*<.001). Physician-patient communication efficacy exhibited a significant negative indirect association with patient adherence (β=–0.026; *P*<.001), accounting for 9.29% of the total association. Platform type significantly moderated this association: internet hospitals (β=0.099; *P*=.04), professional platforms (β=0.081; *P*=.04), and WeChat accounts (β=0.032; *P*=.03) enhanced the positive association between OHISB and patient adherence, whereas short video platforms (β=–0.034; *P*=.006) and search engines (β=–0.204; *P*<.001) weakened it.

**Conclusions:**

Online health information seeking among rural residents was directly associated with better patient adherence, but this benefit was partially attenuated by a negative indirect association through reduced physician-patient communication efficacy. The association between OHISB and adherence varied significantly by platform type. This finding suggests the need for digital health equity strategies, interventions to improve communication efficacy and health literacy, and graded management of health information platforms.

## Introduction

Driven by widespread internet adoption, rural residents’ daily lives are rapidly undergoing digital transformation. By December 2024, internet users in rural China reached 326 million, with a penetration rate of 65.6%, representing a 28.1–percentage point increase since 2018 [1]. Concurrently, online health information seeking has emerged as the primary channel for public acquisition of medical knowledge. This shift has reconfigured patients’ disease awareness and decision-making patterns while also reshaping traditional physician-patient dynamics [2]. Patient adherence, a core metric for evaluating health care outcomes, is increasingly determined by the dynamic interaction between patient autonomy and health literacy rather than physician authority [3].

However, current research predominantly examines the direct pathways from health information seeking to adherence, overlooking the mediating role of physician-patient communication efficacy. In the digital era, patients’ prior knowledge acquired through online sources may compromise communication effectiveness, potentially reducing interaction quality and indirectly impairing adherence [4]. Moreover, heterogeneous health information platforms (eg, professional medical portals, short video platforms, and search engines) may exert differential moderating effects on the information seeking–adherence relationship due to disparities in information quality, credibility, and user experience [5].

To address this gap, we developed a conceptual framework grounded in the health belief model (HBM) [6,7]. This framework posits that online health information–seeking behavior (OHISB) may enhance patient adherence directly by strengthening perceived benefits and self-efficacy. Simultaneously, OHISB may trigger information overload and uncertainty—functioning as perceived barriers within the HBM framework—which may undermine physician-patient communication efficacy and, thereby, exert a negative indirect effect on adherence. Platform type is theorized to moderate these associations by shaping the quality and credibility of the information encountered. Specifically, platforms with rigorous content curation (eg, hospital-affiliated portals) are hypothesized to strengthen the positive OHISB-adherence association, whereas platforms characterized by fragmented, entertainment-oriented, or low-credibility content (eg, short video apps and search engines) are hypothesized to weaken or reverse this association. Accordingly, the primary objective of this study was to examine the association between OHISB and patient adherence among rural Chinese residents, with a specific focus on the mediating role of physician-patient communication efficacy and the moderating role of platform type.

## Methods

### Study Design and Participants

This cross-sectional study was conducted from June 19, 2023, to October 20, 2024. A multistage stratified sampling method was used. First, 2 provinces (autonomous regions and municipalities) were selected from each of the eastern, central, and western regions of China to ensure national geographical representativeness. The surveyed provinces included Gansu, Chongqing, Hubei, Henan, Zhejiang, and Shandong. Within each province, counties were stratified by economic development level; the top and bottom 3% of counties were selected as sampling units, totaling 12 counties.

Trained investigators conducted door-to-door interviews with permanent residents (defined as individuals who had resided in the household for ≥6 months in the previous year), administering paper questionnaires to individuals aged 18 to 70 years. A standardized training manual and interview script were used (Multimedia Appendix 1). All investigators underwent a 2-day centralized training session covering research ethics, questionnaire explanation, and interview techniques. Role-playing and pilot tests were conducted to ensure uniform understanding and delivery of questions. Regular supervision (weekly team meetings to review completed questionnaires) and random callback checks (10% of participants were recontacted via telephone within 1 week to verify responses) were performed during data collection to maintain consistency and quality. The questionnaire was interviewer administered: investigators read each item aloud and recorded responses to minimize literacy-related barriers.

Each questionnaire included a written informed consent form. The study excluded minors. Of the 7200 returned questionnaires, 7004 (97.3%) valid responses were collected after excluding incomplete or inconsistent submissions. Inclusion criteria were (1) medical resource use within the previous 2 years, (2) language communication ability, (3) no major cognitive or mental disorder, and (4) voluntary participation.

### Variables

#### Independent Variable

The independent variable was OHISB. Consistent with the conceptual framework by Mirzaei et al [8], this construct encompasses active retrieval, browsing, and acquisition of health-related information via digital platforms. It was measured via self-reported frequency of internet-based health information seeking using computers or mobile devices. Responses were recorded on a 3-point Likert-type scale: 0=“never,” 1=“occasionally” (1-2 times per month), and 2=“frequently” (≥3 times per month). Response categories were defined in the questionnaire with concrete anchors, and interviewers provided standardized examples (eg, “searching for information about symptoms, treatments, or medications online”). This parsimonious measure was selected for its methodological appropriateness in a large-scale survey with rural residents [9], effectively balancing scientific rigor with practical feasibility by capturing essential behavioral variation while minimizing cognitive load and ensuring high response rates.

#### Dependent Variable

Patient adherence constituted the dependent variable. Adopting the World Health Organization’s conceptualization [10], this construct was operationalized as the extent of congruence between patient behaviors and health care providers’ recommendations. It was measured using a single item asking respondents about their willingness to modify health-related behaviors in accordance with clinical guidance. Responses were recorded on a 5-point scale (1=“not at all willing,” 2=“slightly willing,” 3=“moderately willing,” 4=“very willing,” and 5=“completely willing”).

#### Mediating Variable

Physician-patient communication efficacy served as the mediating variable. Measurement used a 5-point scale to assess patients’ self-perceived competence in articulating needs and understanding medical advice [11]. This scale comprised 2 separate items: one for “articulating health concerns to the doctor” and one for “understanding the doctor’s explanations and recommendations.” Scores were averaged to produce a composite measure. To ensure the relevance and psychometric robustness for the rural population, the scale underwent formal adaptation and validation. Content validity was evaluated by 3 independent experts in health communication (2 PhD-level health communication researchers and 1 practicing physician with ≥10 years of clinical experience), yielding a content validity index greater than 0.85. A pilot study with a separate sample of 30 rural residents (excluded from the final analysis) assessed structural validity and reliability. Exploratory factor analysis of the pilot data supported the presumed unidimensional structure (Kaiser-Meyer-Olkin test=0.805; Bartlett test of sphericity: *P*<.001). In the main study sample (N=7004), the scale demonstrated excellent internal consistency (Cronbach α=0.915).

#### Moderating Variable

The moderating variable was platform type for health information seeking. This construct was operationalized by categorizing respondents’ primary digital sources into five mutually exclusive types: (1) public hospital–affiliated online portals, (2) specialized medical platforms (eg, Haodf or Ping An Good Doctor), (3) short video apps (eg, Douyin, Kuaishou, or RedNote), (4) WeChat official accounts, and (5) search engines (eg, Baidu and Quark).

### Covariates

Potential confounding variables were controlled based on established methodological approaches [12], encompassing age, educational attainment, annual household income, employment status, chronic disease status, self-rated health, weekly breakfast frequency, smoking status, alcohol consumption, exercise frequency, and subjectively measured sleep quality.

### Statistical Methods

Data entry and database development were performed using EpiData (version 4.6; EpiData Association) with double entry verification. All statistical analyses were conducted in R (version 4.3.1; R Foundation for Statistical Computing) using the *lme4* package for fixed-effects modeling and *glmnet* for least absolute shrinkage and selection operator (LASSO) regression, with statistical significance defined as a 2-sided *P* value of less than .05. Baseline regression analyses incorporated regional fixed effects, and SEs were clustered at the county level to account for intracounty correlation, providing more conservative statistical inferences. Robustness checks were implemented through LASSO regression, a machine learning method used for variable selection and shrinkage to prevent overfitting in models with many potential covariates. LASSO helps identify a parsimonious set of predictors by shrinking less important coefficients toward zero, thereby addressing potential multicollinearity.

To examine whether physician-patient communication efficacy mediated the association between OHISB and patient adherence, we conducted a mediation analysis using the bootstrap method with 5000 resamples [13]. The indirect effect (product of the coefficient for OHISB to efficacy and efficacy to adherence) and its 95% CI were calculated. Mediation was considered statistically significant if the CI did not include 0.

### Ethical Considerations

This study was approved by the ethics committee of Tongji Medical College of Huazhong University of Science and Technology (IORG0003571). All procedures performed were in accordance with the ethical standards of the institutional research committee and with the 1964 Declaration of Helsinki and its later amendments. Participation was entirely voluntary and anonymous; no personally identifiable information was collected. All data were kept strictly confidential and used solely for research purposes. Electronic informed consent was obtained from all individual participants included in the study. No financial compensation was provided to the participants.

## Results

### Descriptive Analysis

The final sample included 7004 rural participants. OHISB distribution was 12.4% (867/7004) frequent users, 18.3% (1280/7004) occasional users, and 69.3% (4857/7004) nonusers. Mean scores were 3.43 (SD 1.41) for patient adherence and 4.32 (SD 0.98) for physician-patient communication efficacy. Regarding health information platforms (percentages calculated among OHISB users: 2147/7004, 30.7%), short video apps showed the highest use (1597/2147, 74.4%), followed by WeChat official accounts (575/2147, 26.8%), whereas specialized platforms demonstrated minimal adoption (hospital-affiliated portals: 39/2147, 1.8%; dedicated medical platforms: 47/2147, 2.2%).

Table 1 presents the demographic and health-related characteristics of the 7004 participants, including age, educational attainment, income, employment status, region, chronic disease status, self-rated health, and lifestyle factors (breakfast frequency, smoking, drinking, exercise, and sleep quality).

**Table 1.** Demographic and health-related characteristics of the participants (N=7004).

Variable and category	Values
Age (y), mean (SD)	54.72 (23.81)
Educational level (1-5)^a^, mean (SD)	2.48 (1.13)
Annual income (US $)^b^, mean (SD)	2493.73 (4499.05)
Employment status, n (%)	
Unemployed	3382 (48.3)
Employed (including farming)	3622 (51.7)
Region, n (%)	
Eastern	2394 (34.2)
Central	2552 (36.4)
Western	2058 (29.4)
Chronic diseases, n (%)	
No	4256 (60.8)
Yes	2748 (39.2)
EQ-5D score (−0.149 to 1.000), mean (SD)	0.91 (0.17)
Frequency of breakfast (1-5)^c^, mean (SD)	1.31 (0.99)
Smoking status (1-3)^d^, mean (SD)	1.81 (0.39)
Drinking status (1-3)^e^, mean (SD)	1.81 (0.39)
Weekly physical exercise sessions, mean (SD)	3.59 (3.45)
Sleep quality (1-5)^f^, mean (SD)	2.20 (1.17)

a 1=never attended school; 2=primary school; 3=junior high school; 4=high school; 5=college degree or higher.

b Converted at the average exchange rate of US $1=CN ¥7.1383, mean of the 2023 and 2024 annual average rates from the Federal Reserve.

c 1=daily; 2=4‐6 times per week; 3=1‐2 times per week; 4=once per week; 5=never.

d 1=smoker; 2=quit smoking; 3=nonsmoker.

e 1=drinks alcohol; 2=has given up alcohol; 3=does not drink alcohol.

f 1=verygood; 2=good; 3=average; 4=poor; 5=very poor.

Table 2 summarizes the distribution of the core study variables, including OHISB frequency, patient adherence scores, physician-patient communication efficacy scores, and the use of different platform types for health information seeking (percentages calculated among OHISB users in this case).

**Table 2.** Distribution of core study variables(N=7004).

Variable and category	Values
OHISB^a^ frequency, n (%)	
Never	4857 (69.3)
Occasionally	1280 (18.3)
Frequently	867 (12.4)
Patient adherence (1-5), mean (SD)	3.43 (1.41)
Physician-patient communication efficacy (1-5), mean (SD)	4.32 (0.98)
Platform types for health information seeking (n=2147), n (%)^b^	
Public hospital–affiliated online portals	39 (1.8)
Specialized medical platforms	47 (2.2)
Shortvideo apps	1597 (74.4)
WeChat official accounts	575 (26.8)
Search engines	240 (11.2)

a OHISB: online health information–seeking behavior.

b Percentages may add up to more than 100 due to multiple platform use.

### Association Between OHISB and Patient Adherence

As shown in Table 3, OHISB was significantly and positively associated with patient adherence across all models.

**Table 3.** Association between online health information–seeking behavior (OHISB) and patient adherence (N=7004)^a^.

Variable	Model 1^b^, β (SE)	Model 2^c^, β (SE)	Model 3^d^, β (SE)	Model 4^e^, β (SE)
OHISB	0.356^f^ (0.024)	0.354^f^ (0.024)	0.260^f^ (0.024)	0.268^f^ (0.024)
Age	—^g^	—	0.003^f^ (0.001)	0.005^f^ (0.001)
Educational attainment	—	—	0.116^f^ (0.017)	0.117^f^ (0.016)
Annual income	—	—	0.027^f^ (0.005)	—
Employment status	—	—	0.113^h^ (0.041)	—
Chronic disease	—	—	–0.067 (0.038)	—
Health (EQ-5D)	—	—	0.962^f^ (0.106)	0.876^f^ (0.102)
Breakfast frequency	—	—	–0.103^f^ (0.017)	–0.101^f^ (0.017)
Smoking	—	—	–0.029 (0.048)	—
Drinking	—	—	0.139^h^ (0.048)	—
Exercise frequency	—	—	0.040^f^ (0.005)	0.042^f^ (0.005)
Sleep quality	—	—	0.020 (0.015)	—

a Regional fixed effects were applied at the province level for models 2, 3, and 4. Statistical significance was assessed using linear regression with 2‑tailed *t *tests.

b Unadjusted; *R*2=0.496; adjusted *R*2=0.496; root mean squared error (RMSE)=1.39.

c Regional fixed effects; *R*2=0.532; adjusted *R*2=0.520; RMSE=1.38.

d All covariates (full list in the Methods section); *R*2=0.587; adjusted *R*2=0.573; RMSE=1.35.

e Least absolute shrinkage and selection operator–selected covariates; *R*2=0.542; adjusted *R*2=0.541; RMSE=1.35.

f *P*<.001.

g Variable not included in the model.

h *P*<.01.

In the unadjusted model (model 1), the association was strong (β=0.356; *P*<.001). Incorporating regional fixed effects in model 2 yielded consistent estimates (β=0.354; *P*<.001), confirming model robustness. Regional fixed effects were applied at the province level. Model 3 further adjusted for covariates, including age, income, and educational attainment, maintaining statistical significance (β=0.260; *P*<.001). This corresponds to a 0.260-unit increase in adherence per 1-unit increment in OHISB frequency. Significant covariates associated with higher adherence included older age, higher educational attainment, better self-rated health, consistent breakfast habits, and greater exercise frequency.

Model 4 used LASSO regression for robustness checks. The regularization procedure retained significant covariates: age, educational level, health (EQ-5D), weekly frequency of breakfast, and exercise frequency. OHISB showed a consistent positive association with patient adherence (β=0.268; *P*<.001) relative to fixed-effects models. Control variable coefficients maintained directional and statistical alignment with baseline estimates.

The results of the 4 models demonstrated the robustness of the association between OHISB and patient adherence.

### Mediation Analysis

To examine the mechanism through which OHISB may be associated with patient adherence, a mediation analysis was conducted using the bootstrap method with 5000 resamples. The results, presented in Table 4, delineate the direct, indirect, and total associations.

**Table 4.** Mediation analysis of physician-patient communication efficacy^a^.

Path	Estimate (β; SE; 95% CI)	*P* value
a: OHISB^b^–efficacy	−0.083 (0.018; –0.117 to –0.048)	<.001
b: efficacy–adherence	0.310 (0.016; 0.278 to 0.342)	<.001
c’: direct (OHISB–adherence)	0.301 (0.024; 0.255 to 0.348)	<.001
Indirect (a × b)	−0.026 (0.007; –0.039 to –0.013)	<.001
Total effect (c)	0.276 (0.024; 0.228 to 0.324)	<.001

a Bootstrap resamples=5000; proportion of total effect mediated: 9.29%; *P* value for indirect effect based on bootstrap percentile method.

b OHISB: online health information–seeking behavior.

Path *a* (OHISB–communication efficacy) was negative and significant (β=−0.083; *P*<.001, 95% CI −0.117 to –0.048). Path *b* (communication efficacy–adherence) was positive and significant (β=0.310; *P*<.001, 95% CI 0.278-0.342). The indirect effect (*a* × *b*) was negative and significant (β=−0.026; *P*<.001, 95% CI −0.039 to −0.013), accounting for 9.29% of the total association. The direct effect (*c*’: OHISB–adherence, controlling for efficacy) remained positive and significant (β=0.301; *P*<.001, 95% CI 0.255-0.348), indicating inconsistent mediation.

**Figure 1.**
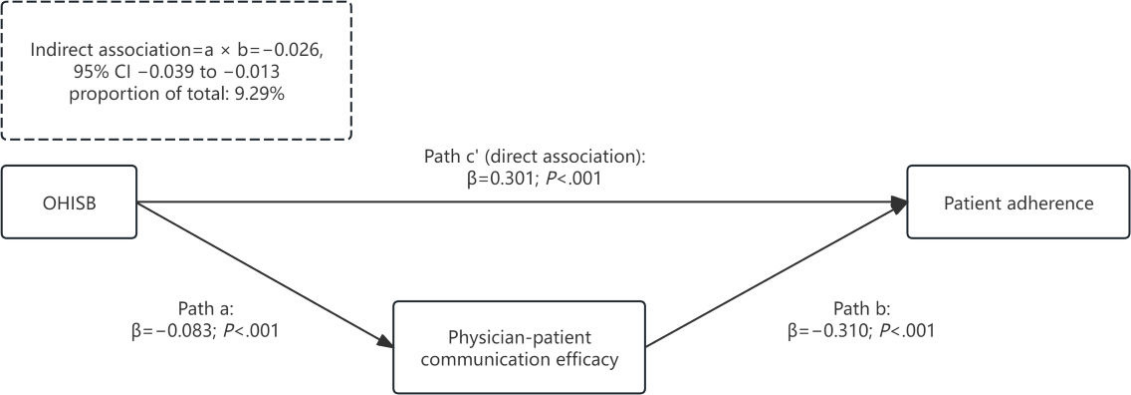
Mediation model of physician-patient communication efficacy in the association between online health information–seeking behavior (OHISB) and patient adherence.

This indirect association accounted for approximately 9.29% of the total association between OHISB and adherence, indicating an inconsistent mediation pattern (ie, the indirect association opposes the direction of the direct positive association) [13,14]. Figure 1 shows the mediation model with standardized path coefficients.

### Moderation by Platform Type

All interaction terms attained statistical significance (Table 5). Positive moderating associations were observed for hospital-affiliated portals (β=0.099; *P*=.04), specialized medical platforms (β=0.081; *P*=.04), and WeChat official accounts (β=0.032; *P*=.03), with hospital-affiliated portals showing the largest effect size. Negative moderating associations were observed for short video apps (β=−0.034; *P*=.006) and search engines (β=−0.204; *P*<.001), where search engines demonstrated the most pronounced attenuating pattern.

**Table 5.** Moderating roles of platform type on the relationship between online health information–seeking behavior (OHISB) and adherence (N=7004)^a^.

Variable	Model 1^b^, β (SE)	Model 2^c^, β (SE)	Model 3^d^, β (SE)	Model 4^e^, β (SE)	Model 5^f^, β (SE)
OHISB	0.061^g^ (0.006)	0.063^g^ (0.006)	0.022^h^ (0.011)	0.055^g^ (0.007)	0.062^g^ (0.006)
Hospital-affiliated portals	0.013 (0.184)	—^i^	—	—	—
OHISB × hospital-affiliated portals	0.099^h^ (0.044)	—	—	—	—
Specialized medical platforms	—	0.052 (0.132)	—	—	—
OHISB × specialized medical platforms	—	0.081^h^ (0.035)	—	—	—
Short video apps	—	—	−0.265^g^ (0.043)	—	—
OHISB × short video apps	—	—	−0.034^j^ (0.012)	—	—
WeChat official accounts	—	—	—	0.074 (0.045)	—
OHISB × WeChat official accounts	—	—	—	0.032^h^ (0.013)	—
Search engines	—	—	—	—	0.298^g^ (0.066)
OHISB × search engines	—	—	—	—	−0.204^g^ (0.007)

a All models control for the covariates listed in Table 3. The reference category for each interaction term is nonuse of the respective platform type.

b *R*2=0.642; adjusted *R*2=0.625; root mean squared error (RMSE)=0.66.

c *R*2=0.637; adjusted *R*2=0.615; RMSE=0.66.

d *R*2=0.592; adjusted *R*2=0.583; RMSE=0.64.

e *R*2=0.583; adjusted *R*2=0.577; RMSE=0.66.

f *R*2=0.639; adjusted *R*2=0.621; RMSE=0.65.

g *P*<.001.

h *P*<.05.

i Variable not included in the model.

j *P*<.01.

The reference category for each interaction term was nonuse of the respective platform type. For example, the coefficient for OHISB × short video apps (β=−0.034) indicates that, compared with nonusers of short video platforms, users of these platforms showed a weaker positive association between OHISB and adherence.

## Discussion

This study revealed a dual-process association between OHISB and patient adherence among rural Chinese residents moderated by the type of digital platform used.

### Positive Association Between OHISB and Adherence Amid Low Adoption Rates

OHISB frequency was positively associated with patient adherence, consistent with prior research [15,16]. This finding supports the HBM [17], suggesting that information seeking may enhance perceived treatment benefits and self-efficacy, directly promoting adherence intentions. The adoption of OHISB is significantly lower in rural populations compared with reported rates in urban settings, and the joint use of hospital-affiliated platforms and professional health information platforms is relatively low in these populations [2,18]. This disparity likely stems from overlapping digital divides: deficient rural infrastructure, technological barriers among older adults, and terminology-dense content that raises cognitive thresholds [19]. The high health literacy demands of professional platforms further limit their potential benefits [20]. Future initiatives must optimize platform usability and deliver comprehensible, tailored health messaging to rural populations.

### The Dual-Process Model: Unpacking the Negative Indirect Association

Our analysis suggests a dual-process pattern: while OHISB was directly and positively associated with adherence, a simultaneous negative indirect association was observed through reduced physician-patient communication efficacy [21]. This finding must be interpreted with nuance; it does not imply that online information seeking is inherently harmful [22]. Rather, it highlights a critical contingency: the net benefit of OHISB may be weaker when the information environment or the clinical encounter creates specific risks. Reduced communication efficacy may be more likely under circumstances where (1) information from online sources is fragmented, contradictory, or of low credibility, which may contribute to patient confusion or misplaced confidence [23]; (2) patients experience “information overload,” a state that increases anxiety and hinders effective information processing during consultations [23]; or (3) a power dynamic is activated wherein clinicians perceive patient-initiated information as challenging their authority, prompting defensive communication [24]. For health system design, these findings suggest that promoting patient empowerment through digital tools should be coupled with parallel interventions to improve information quality; scaffold patient-clinician dialogue; and train clinicians in collaborative, information-integrated consultation styles. The indirect effect, while statistically significant, accounted for only 9.29% of the total association, suggesting that the direct positive association predominated.

### Platform-Specific Differences in the OHISB-Adherence Association

The moderating effects of platform types exhibited significant heterogeneity. Hospital-affiliated portals, specialized medical platforms, and WeChat official accounts strengthened the positive association between OHISB and patient adherence, whereas short video apps and search engines weakened it, corroborating previous findings [25]. Mechanistically, this divergence likely stems from differential information credibility, content quality, and interaction paradigms. Hospital-affiliated and specialized platforms enforce rigorous content curation to deliver evidence-based structured information congruent with clinical standards, enabling patients to integrate information into shared decision-making [26]. WeChat official accounts further facilitate sustained physician-patient interaction that contextualizes health information. Conversely, despite dominating use in this study (1597/2147, 74.4%), short video and search platforms propagate fragmentary, entertainment-focused content (eg, treatment claims emphasizing efficacy while omitting risks). Their recommendation algorithms may create echo chambers that increase exposure to homogeneous viewpoints and amplify cognitive biases [24]. Concurrently, their attention-grabbing formats trivialize complex health topics, whereas inconsistent information quality exposes users to contradictory perspectives, generating information overload [22]. This complexity may induce decisional uncertainty that ultimately attenuates the benefits of information seeking.

However, the observed platform-specific differences should be interpreted with caution as they may partly reflect unmeasured confounding factors. For instance, users of different platforms may differ systematically in digital literacy, health literacy, access to alternative information sources, or underlying health status. Short video platforms, which dominate rural use, may attract users with lower digital literacy or less engagement with formal health care, whereas those who actively seek out hospital-affiliated portals may have higher health literacy or more complex health needs. Such self-selection could contribute to the differential associations observed. Future research should measure and adjust for these potential confounders to isolate the unique moderating effects of platform characteristics.

### Limitations

This study has several limitations. First, its cross-sectional design precludes causal inference; longitudinal or experimental studies are needed to establish temporal sequences and strengthen causal claims. Consequently, terms such as “effect,” “impact,” and “pathway” are used descriptively to reflect statistical associations rather than causal relationships. Second, the use of a single-item, 3-point scale to measure OHISB, while practical, lacks granularity and conceptual specificity. Third, patient adherence was measured using a single item assessing behavioral intention rather than actual behavior, which may overestimate adherence due to social desirability bias. Test-retest reliability was not assessed, which represents an additional limitation. Future work should use validated, multidimensional scales to capture this complexity and provide a more nuanced understanding of the OHISB and patient adherence construct.

### Conclusions

Drawing on cross-sectional survey data collected during 2023 and 2024, this study investigated OHISB and patient adherence, with particular emphasis on the mediating role of physician-patient communication efficacy and moderating associations with platform type. Three principal findings emerged.

First, OHISB frequency was positively associated with patient adherence, wherein higher seeking frequency corresponded to greater adherence. Second, physician-patient communication efficacy was observed as a negative indirect association whereby OHISB was associated with reduced communication efficacy, which in turn was associated with lower adherence—partially offsetting the direct positive association. However, this indirect association accounted for only 9.29% of the total association, indicating that the direct positive association predominated. Third, the association between information seeking and adherence varied significantly by platform type: hospital-affiliated portals, specialized medical platforms, and WeChat official accounts showed positive moderating associations, whereas short video apps and search engines showed attenuating associations that diminished overall empowerment returns.

These findings align with the HBM. The direct positive association supports the HBM’s premise that information seeking enhances perceived benefits and self-efficacy. The negative indirect association is consistent with the concept of perceived barriers: information overload and uncertainty may impede effective patient-clinician interaction. Platform-specific moderation suggests that information quality and credibility shape whether seeking behavior reduces or amplifies these barriers.

These findings suggest several implications for policy and practice, although these should be considered suggestive rather than definitive given the observational study design. First, to leverage the direct positive association and address low adoption rates, digital health equity initiatives should include the co-design of culturally and linguistically adapted health information content (eg, developing plain-language, dialect-compatible health materials for low-literacy users) with rural residents. Second, to address the negative indirect association, health systems might consider dual-focused interventions: (1) for patients, brief, scalable “health information literacy” modules to build skills for critical evaluation of online content; and (2) for clinicians, training in communication strategies to acknowledge, discuss, and integrate patient-sourced information constructively. Third, to respond to the heterogeneous platform effects, a graded regulatory framework could be considered, including accreditation incentives for authoritative content on professional platforms and algorithmic governance measures for short video and search platforms (eg, mandatory source disclosure for health claims and downgrading of unverified medical advice).

These findings and implications are grounded in a rural Chinese context and may not be directly generalizable to other settings. Future research should use longitudinal designs, validated multidimensional measures, and diverse populations to extend and refine these observations.

## Supplementary material

10.2196/91115 Multimedia Appendix 1 Interviewer training manual and interview script.

## Abbreviations

HBM: health belief model
LASSO: least absolute shrinkage and selection operator
OHISB: online health information–seeking behavior
