# Supplementary material for: Associations and Pathways Between Online Health Information–Seeking Behavior and Patient Adherence: Cross-Sectional Study
Source: J Med Internet Res. 2026 Jun 4;28:e91115. doi: 10.2196/91115 (PMC13235974; doi:10.2196/91115)
Supplement: Multimedia Appendix 1 [file jmir-v28-e91115-s001.pdf]

访员培训手册

一、培训目的

确保所有访员统一理解研究目的、问卷内容、访谈流程和伦理规范，保证数据收集的标准化和高质量。

二、培训内容

2.1 研究背景与目的

本研究旨在了解农村居民在线健康信息寻求行为与患者依从性的关系。

调查对象为 18-70 岁农村常住居民（过去一年内在该家庭居住≥6 个月）。

数据将用于学术研究，严格保密。

2.2 伦理规范

每位受访者必须签署知情同意书。

向受访者说明：自愿参与，可随时退出，无任何不良后果。

不得泄露任何受访者个人信息。

2.3 问卷结构与说明

问卷共 17 题，分为五个部分。

所有问题均使用标准化措辞，不得自行解释或引导。

对于 Likert 量表题，需向受访者清晰展示选项卡。

2.4 关键变量定义与锚点

变量	选项	定义/锚点

变量	选项	定义/锚点
OHISB 频率	0=从不	不使用互联网查找健康信息
	1=偶尔	每月 1-2 次
	2=经常	每月 3 次及以上
患者依从性	1-5	1=完全不愿意 → 5=非常愿意
医患沟通效能	1-5	1=完全不能 → 5=完全能

2.5 访谈流程

敲门、自我介绍、说明来意。

确认受访者资格（年龄 18-70 岁、常住居民、过去两年内使用过医疗服务）。

获得知情同意。

逐题朗读问题，记录回答。

检查问卷完整性，致谢。

2.6 角色扮演练习

访员两两配对，模拟完整访谈过程。

练习如何处理受访者的疑问（如“我不太明白这个问题”）。

练习如何应对拒绝或中断。

2.7 质量控制

定期监督：每周召开团队会议，回顾已完成的问卷，讨论问题。

随机回访检查：抽取 10%的受访者，在一周内通过电话回访，验证关键信息（如年龄、在线信息搜寻频率）。

三、常见问题处理

问题	标准回应
“这个调查是干什么的？”	“我们正在做一个关于农村居民健康信息获取和就医行为的学术研究，您的回答会帮助我们更好地了解农村健康需求。”
“我能不能不回答某个问题？”	“当然可以。您有权跳过任何您不愿意回答的问题。”
“这个问题是什么意思？”	（按照培训手册中的标准解释进行说明，不得自行发挥）

Interviewer Training Manual

1. Training Objectives

To ensure that all interviewers have a unified understanding of the research objectives, questionnaire content, interview procedures, and ethical standards, thereby ensuring standardized and high-quality data collection.

2. Training Content

2.1 Research Background and Objectives

This study aims to understand the association between online health information-seeking behavior and patient adherence among rural residents.

Target population: Rural permanent residents aged 18-70 years (defined as having resided in the household for ≥6 months in the past year).

Data will be used for academic research and will be kept strictly confidential.

2.2 Ethical Standards

Each participant must sign an informed consent form.

Inform participants that participation is voluntary, they may withdraw at any time, and there will be no negative consequences.

Do not disclose any personal information of participants.

2.3 Questionnaire Structure and Instructions

The questionnaire has 17 items divided into five sections.

All questions must be read using standardized wording; do not paraphrase or lead the participant.

For Likert-scale questions, clearly show the participant the response card.

2.4 Key Variable Definitions and Anchors

Variable	Response	Definition / Anchor
OHISB frequency	0 = Never	Does not use the internet to search for health information
	1 = Occasionally	1-2 times per month
	2 = Frequently	≥3 times per month
Patient adherence	1-5	1 = Not at all willing → 5 = Completely willing
Doctor-patient communication efficacy	1-5	1 = Not at all → 5 = Completely

2.5 Interview Procedure

Knock on door, introduce yourself, state the purpose of the visit.

Confirm participant eligibility (age 18-70, permanent resident, used healthcare services in past 2 years).

Obtain informed consent.

Read each question aloud and record responses.

Check questionnaire completeness and thank the participant.

2.6 Role-Playing Exercises

Interviewers pair up to simulate the full interview process.

Practice handling participant questions (e.g., "I don't quite understand this question").

Practice responding to refusals or interruptions.

2.7 Quality Control

Regular supervision: Weekly team meetings to review completed questionnaires and discuss any issues.

Random callback checks: 10% of participants re-contacted by telephone within one week to verify key information (e.g., age, OHISB frequency).

3. Handling Common Questions

Question	Standard Response
"What is this survey for?"	"We are conducting an academic study on rural residents' health information seeking and healthcare behavior. Your

Question	Standard Response
	answers will help us better understand rural health needs.”
“Can I skip a question?”	“Of course. You have the right to skip any question you are not comfortable answering.”
“What does this question mean?”	(Provide the standardized explanation from the training manual; do not improvise.)

访谈脚本

（开场白）

访员："您好！我是华中科技大学的调查员。我们正在进行一项关于农村居民健康信息获取与就医行为的科学研究。您的参与将帮助我们更好地了解农村健康需求。本调查大约需要 10-15 分钟。您是否愿意参加？"

（如果同意）

访员："非常感谢。我将先向您说明几点：第一，您的回答将被严格保密，仅用于学术研究；第二，您有权随时退出或不回答任何问题；第三，所有问题没有对错之分，请根据您的真实情况回答。请问我可以开始了吗？"

（如果同意）

（知情同意）

访员："请您阅读并签署这份知情同意书。如果您不识字，我可以为您朗读。"

（确保签署后，开始正式访谈）

（正式访谈）

访员："现在我开始提问。我会逐题朗读，请您根据您的实际情况回答。"

第一部分：基本信息

“请问您的年龄是多少？” → 记录：______ 岁

“您目前居住在哪个省？哪个市或县？” → 记录：______ 省 ______ 市/县

“请问您的文化程度是？” → 展示选项卡：1 未上过学 2 小学 3 初中 4 高中/中专 5 大专及以上 → 记录

“请问您家去年的年收入大约是？” → 记录：______ 元

“请问您目前有工作吗？包括务农。” →0 无业 1 有工作 → 记录

“请问您是否有慢性疾病，比如高血压、糖尿病、冠心病等？” →0 否 1 是（请说明）→ 记录

“总体而言，您认为您目前的健康状况如何？”（EQ-5D）→ 访员根据受访者回答记录分值

“您通常每周吃早餐的频率是？” → 展示选项卡 → 记录

“请问您的吸烟情况是？” → 展示选项卡 → 记录

“请问您的饮酒情况是？” → 展示选项卡 → 记录

“您每周平均进行体育锻炼多少次？” → 记录：______ 次/周

“您认为您的睡眠质量如何？” → 展示选项卡 → 记录

第二部分：在线健康信息寻求行为

“您是否使用电脑或手机上网查找健康相关的信息？比如疾病症状、治疗方法、药物信息等。” → 展示选项卡：0 从不 1 偶尔（每月 1-2 次）2 经常（每月 3 次及以上）→ 记录

（如果回答“0 从不”，跳至依从性询问。）

“您最常使用哪些平台获取健康信息？可以多选。”→ 展示选项卡：1 公立医院官方平台 2 专业医疗平台 3 短视频应用 4 微信公众号 5 搜索引擎 6 其他 → 记录所有勾选

第三部分：患者依从

“请问您是否愿意按照医生的建议改变自己的健康相关行为？比如按时吃药、调整饮食、增加运动等。” → 展示选项卡：1 完全不愿意 2 不太愿意 3 一般 4 比较愿意 5 非常愿意 → 记录

第四部分：医患沟通效能

“请您根据最近一次就医的经历回答。第一，您是否能够清晰地向医生表达自己的健康问题和担忧？” → 展示选项卡：1 完全不能 2 不太能 3 一般 4 比较能 5 完全能 → 记录

“第二，您是否能够理解医生对病情、治疗方案和注意事项的解释？” → 展示选项卡同上 → 记录

第五部分：就医情况

“过去两年内，您是否使用过医疗服务？比如门诊、住院、体检等。” →0 否 1 是 → 记录

（结束语）

访员：“所有问题已经问完了。非常感谢您的宝贵时间和真诚回答！您的参与对我们的研究非常重要。祝您身体健康！”

（检查问卷是否完整，是否有漏填项）

Interview Script

(Opening)

Interviewer: “Hello! I am an interviewer from Huazhong University of Science and Technology. We are conducting a scientific study on rural residents’ health information seeking and healthcare behavior. Your participation will help us better understand rural health needs. This survey will take about 10-15 minutes. Would you be willing to participate?”

(If yes)

Interviewer: “Thank you. Let me first explain a few things: First, your answers will be kept strictly confidential and used only for academic research. Second, you have the right to withdraw at any time or skip any question. Third, there are no right or wrong answers—please answer based on your true situation. May I begin?”

(If yes)

(Informed Consent)

Interviewer: “Please read and sign this informed consent form. If you cannot read, I can read it to you.”

(After obtaining signature, begin formal interview)

(Formal Interview)

Interviewer: “I will now begin the interview. I will read each question

aloud. Please answer based on your actual situation.”

Part 1: Demographic Information

“May I ask your age?” → Record: ______ years

“Which province and city/county do you currently live in?” → Record: ______ Province ______ City/County

“What is your highest education level?” → Show response card: 1 Never attended school 2 Primary school 3 Junior high school 4 High school/vocational 5 College or above → Record

“What was your household’s total annual income last year?” → Record: ______ RMB

“Are you currently employed? Including farming.” → 0 Unemployed 1 Employed → Record

“Do you have any chronic disease, such as hypertension, diabetes, or coronary heart disease?” → 0 No 1 Yes (specify) → Record

“Overall, how would you rate your current health status?” (EQ-5D) → Interviewer records score based on participant’s response

“How often do you eat breakfast per week?” → Show response card → Record

“What is your smoking status?” → Show response card → Record

“What is your drinking status?” → Show response card → Record

“How many times per week do you exercise on average?” → Record:

______ times/week

“How would you rate your sleep quality?” → Show response card → Record

Part 2: Online Health Information Seeking Behavior

“Do you use computers or mobile devices to search for health-related information online? For example, symptoms, treatments, medication information.” → Show response card: 0 Never 1 Occasionally (1-2 times/month) 2 Frequently (≥3 times/month) → Record

(If “0 Never”, skip to ask patient adherence.)

“Which platforms do you most commonly use to obtain health information? You may select more than one.” → Show response card: 1 Hospital portals 2 Specialized medical platforms 3 Short-video apps 4 WeChat Official Accounts 5 Search engines 6 Other → Record all selected

Part 3: Patient Adherence

“Are you willing to modify your health-related behaviors—such as taking medication, adjusting your diet, or increasing exercise—in accordance with your doctor’s recommendations?” → Show response card: 1 Not at all willing 2 Slightly willing 3 Moderately willing 4 Very willing 5 Completely willing → Record

Part 4: Doctor-Patient Communication Efficacy

“Please answer based on your most recent healthcare visit. First, can you clearly articulate your health concerns and questions to the doctor?” → Show response card: 1 Not at all 2 Slightly 3 Moderately 4 Very much 5 Completely → Record

“Second, can you understand the doctor’s explanations of your condition, treatment plan, and precautions?” → Same response card → Record

Part 5: Healthcare Utilization

“Have you used any healthcare services in the past two years? For example, outpatient visits, hospitalization, physical examinations.” → 0 No 1 Yes → Record

---

(Closing)

Interviewer: “That is all the questions. Thank you very much for your time and honest answers! Your participation is very important to our research. I wish you good health!”

(Check questionnaire for completeness and missing items)
